# Effects of a Low-Molecular-Weight Gelator in Vegetable, Mineral Oil and Cocoa Butter: A Comparative Rheological Study

**DOI:** 10.3390/gels12060482

**Published:** 2026-06-01

**Authors:** Emmanuel Anegbe, Cesare Oliviero Rossi, Iolinda Aiello, Nicolas Godbert, Eugenia Giorno, Darren A. Makeiff, Pietro Calandra, Paolino Caputo

**Affiliations:** 1 Dipartimento di Chimica e Tecnologie Chimiche, Università della Calabria, UdR INSTM della Calabria, Via P. Bucci, Cubo 14/D, 87036 Rende, CS, Italy; emmanuel.anegbe@tnuni.sk (E.A.); cesare.oliviero@unical.it (C.O.R.); paolino.caputo@unical.it (P.C.); 2 MAT-InLAB, LASCAMM CR-INSTM, Unità INSTM della Calabria, Dipartimento di Chimica e Tecnologie Chimiche, Università della Calabria, Via P. Bucci 14/C, 87036 Rende, CS, Italy; eugenia.giorno@unical.it; 3 LPM-Laboratorio Preparazione Materiali, Star-Lab, Università della Calabria, 87036 Rende, CS, Italy; 4 CNR NANOTEC, UOS Rende c/o Dipartimento di Fisica, Università della Calabria, Via P. Bucci 33/C, 87036 Rende, CS, Italy; 5 Quantum and Nanotechnologies Research Centre, National Research Council Canada, 11421 Saskatchewan Drive, Edmonton, AB T6G 2M9, Canada; 6 CNR-ISMN, National Research Council, Institute of Nanostructured Materials, Strada Provinciale 35 D n.9, 00010 Montelibretti, RM, Italy

**Keywords:** low-molecular-weight gelator, isophthalic and gallic acid derivatives, organogels, viscosity modifiers, supergelator

## Abstract

The demand for eco-friendly viscosity modifiers in food, cosmetics, and lubricants has increased, promoting the development of high-performance, sustainable materials. Low-molecular-weight gelators (LMWGs) are promising candidates, though their behavior in complex systems remains underexplored. In this study, a novel alkylamido isophthalic acid-based LMWG (AIPA–gallic acid) was synthesized. Its performance was evaluated in vegetable oil, mineral oil, and cocoa butter using rheological measurements across varying concentrations and temperatures, with all dynamic rheological measurements conducted in the viscoelastic region. Cacao butter is solid at 15 °C, so the flow curve that can be obtained at this temperature should show high values not comparable with the other liquid oils. No slippage phenomenon was observed. Using a step-rate protocol before acquiring the flow curves, no time-dependent behavior (thixotropy) was observed. Frequency and flow sweep tests were used to assess viscoelastic properties, interaction strength, and coordination number. Results revealed that incorporating AIPA–gallic acid at 4 wt% increased the viscosity by 74 times (at 25 °C) in mineral oil, compared to an increase of about four orders of magnitude in vegetable oil. This suggests the formation of intermolecular interactions that lead to an increased momentum transport process, which is significantly higher in vegetable oil. In contrast, cocoa butter exhibited minimal rheological changes, suggesting that no gelation occurred. Analysis using the weak gel model confirmed that viscosity enhancement arises from a structured network in mineral and vegetable oils, but not in cocoa butter. Temperature-dependent variations in structural parameters further highlight the role of molecular interactions between the gelator and the oil matrix.

## 1. Introduction

The rheological properties of fats and oils are critical determinants in the formulation of high-performance products across diverse industries, including food, pharmaceuticals, cosmetics, and lubricants. These properties govern not only texture and sensory attributes but also processing behavior, structural stability, and long-term storage performance [1,2]. Increasing regulatory scrutiny and consumer demand for sustainable, health-conscious alternatives have intensified the search for structuring agents capable of modifying oil rheology without relying on saturated or synthetic additives [3,4]. Organogels represent a promising solution [5], where a gelator immobilizes the liquid phase by forming a three-dimensional network, even at relatively low concentrations. The fundamental principles governing organogel formation and structure have been extensively described in seminal works on molecular gels, which highlight the importance of solvent–gelator interactions, thermodynamics, and network morphology in determining macroscopic properties [6,7].

A particularly important class of gelators used in organogel formation is low-molecular-weight gelators (LMWGs), small organic molecules able to self-assemble into supramolecular networks through non-covalent interactions, such as hydrogen bonding, π–π stacking, and van der Waals forces [8]. Because of their small size and reversible interactions, LMWGs offer precise tunability and adaptability across chemically different oil systems. These systems belong to the broader class of molecular gels characterized by self-assembled fibrillar networks, whose structural features and formation mechanisms have been comprehensively discussed in foundational and edited volumes on the subject [9,10].

LMWGs create supramolecular gels through reversible, non-covalent interactions that organize into dynamic, self-assembled fibrous networks capable of trapping solvent molecules. These networks often display thixotropic behavior, meaning that the gel structure temporarily breaks down when subjected to shear stress but reforms once the stress is removed. This unique property stems from the reversible and mobile nature of the non-covalent forces driving gel formation, allowing the network to reorganize itself at the nanoscale [11]. Thixotropy, often referred to as self-healing [12], is well-documented in many low-molecular-weight gelator (LMWG) systems. In these materials, the gel can transition reversibly between gel and sol states, allowing it to recover its mechanical strength after being disrupted by shear [13]. However, this behavior is not universal among supramolecular gels. The ability to recover efficiently depends on several factors, including how soluble the gelator is, how quickly molecular components can exchange or reorganize, and whether any kinetic barriers hinder the reassembly process. Notable examples include peptide- and amino acid-based gels, which demonstrate rapid structural recovery following shear, confirming the ability of LMWG networks to re-form spontaneously under appropriate conditions [14].

Certain LMWGs, termed “supergelators,” exhibit remarkable efficiency at inducing gelation in a wide range of organic solvents, though predicting their behavior remains challenging due to the complex interplay of molecular structure, solvent properties, and kinetics [15,16]. Despite decades of research, the predictive understanding of gelation remains an open challenge, as emphasized in critical perspectives on the evolution and future directions of the field [17]. The versatility of LMWG-based organogels has led to broad applications. In food science, they serve as alternatives to conventional solid fats, enabling reduced saturated fat content while preserving desirable mouthfeel and stability in products such as spreads, dressings, and baked goods [3,4,15].

In cosmetics, LMWGs function as rheology modifiers and carriers for hydrophobic actives, enhancing both product stability and skin penetration [16,18]. In pharmaceuticals, formulations leverage their tunable porosity and viscoelasticity for controlled drug release and wound management [19]. Finally, in lubricants, LMWGs provide thermal resistance, prevent oil migration, and improve durability under mechanical stress [20]. Such versatility is similar in polymeric systems where molecular weight and crosslinking density strongly influence rheological performances [21]. More broadly, such materials fall within the category of soft fibrillar materials, whose fabrication strategies and multifunctional applications have been widely explored in the context of supramolecular and soft matter science [22].

Among LMWGs, alkylamido isophthalic acid (AIPA) derivatives are particularly noteworthy due to their rigid aromatic cores and hydrophobic substituents, which facilitate ordered stacking in organic solvents and efficient self-assembly into gel networks, imparting solid-like properties to the system [23,24]. Their adaptability to both polar and non-polar environments is a key asset when formulating across chemically diverse oil systems. Rheological studies have demonstrated that AIPA-based gelators exhibit a pronounced increase in elastic modulus (G′) with solvent polarity, ranging from 40 Pa in decalin to over 1300 Pa in cyclohexane [24]. However, the integration of LMWGs into complex or crystalline oil systems—such as cocoa butter, a polymorphic triacylglyceride with intricate crystallization behavior—presents significant challenges [24]. Previous work has shown that oil polarity and molecular complexity profoundly influence gel network structure and efficacy [3,25]. Studies involving monoglyceride- and fatty alcohol-based gels report that crystallization onset, hardness, and morphology were strongly influenced by the base oil. Similar conclusions have been drawn by thermorheological investigations of cocoa butter alternatives, suggesting that matrix composition has very sensitive impacts on organogel performance, especially when crystalline states dominate the system [18,25].

To the best of our knowledge, to date, comparative studies that examine LMWG behavior across chemically and structurally diverse oils under consistent experimental conditions are lacking. Most investigations focus on single oil systems, limiting the broader applicability of their findings [26].

Also, gelator performance in complex or crystalline matrices, such as cocoa butter, remains unexplored. Challenges arise from competing interactions between the gelator and the oil’s native structure, which can disrupt network formation [25]. For instance, in triglyceride-rich systems, polymorphic crystallization kinetics may dominate over gelator self-assembly, limiting gelation efficacy [27].

**Figure 1:**
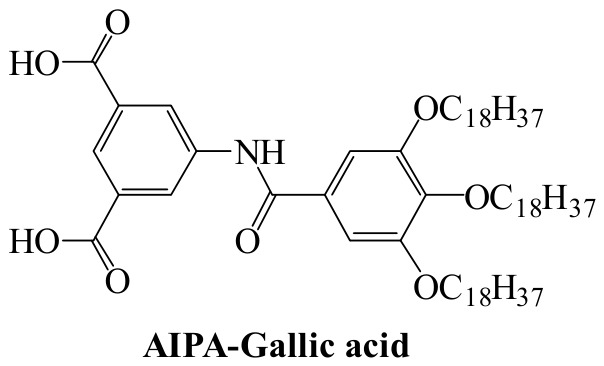
The molecular structure of the AIPA–gallic acid.

This study addresses this gap by systematically analyzing the gelation performance of a novel alkylamido isophthalic acid-based LMWGs (AIPA–gallic acid) bearing a substituted gallic acid fragment with three C_18_ alkyl chains (Figure 1), ad hoc synthesized in our laboratories. The choice of this fragment was originated by our hypothesis that long and alkyl chains could synergically help the aromatic ring in self-assembly giving gelation. It was tested in three oils representing a gradient of structural complexity: mineral oil (Nypar 300), vegetable oil (sunflower oil), and cocoa butter. Mineral oil, with its saturated hydrocarbon content and Newtonian flow behavior, provides a straightforward medium for observing gelator interactions. Vegetable oil, primarily composed of unsaturated triglycerides, presents intermediate complexity, while cocoa butter, with its polymorphic crystallinity, poses the most significant structural challenges to gelation. AIPA–gallic acid was selected as a potential gelator based on the documented efficiency of AIPA-based gelators and their derivatives in inducing gelation across a wide range of apolar solvents at very low concentrations [24]. However, in contrast to previously reported systems featuring linear or branched alkyl chains, a gallic acid fragment functionalized with three octadecyloxy long chains was introduced to enhance compatibility with oil-based matrices.

Using steady-state and oscillatory rheometry, the impact of gelator concentration (0–4 wt%) and temperature (15–65 °C) on key rheological parameters were assessed, including complex modulus (G*), storage/loss moduli (G′, G″), coordination number (z), and interaction strength (A). Insights from the weak gel model elucidate the underlying network formation and stability. Given the comparisons with hydrogels systems based on low-molecular-weight gellan gum, which also exhibit similar gelation behavior influenced by molecular size and crosslinking density, insights from both systems may be mutually informative [21]. Beyond their industrial relevance, these systems may be explored for phase-selective oil recovery or dye entrapment applications, as shown in prior work on isophthalic acid-based supergelators [28].

## 2. Results and Discussions

### 2.1. Preparation of AIPA–Gallic Acid and Gelation Properties

The AIPA–gallic acid was obtained starting from the tris-substituted octadecyl gallic acid (C_18_–gallic acid) whose synthesis was previously described by our group [29]. Formation of the acid chloride derivative of C_18_–gallic acid was performed through reaction with thionyl chloride in dry dichloromethane. The subsequent reaction of gallic acid chloride with the dimethyl 5-aminoisophthalate allows the formation of the amidic bond between the gallic and the isophthalic fragments. Finally, AIPA–gallic acid was obtained by saponification followed by acidification of the thus obtained diacid derivative. Details of all the synthetic steps and compound characterization data are reported in ESI.

**Figure 2:**
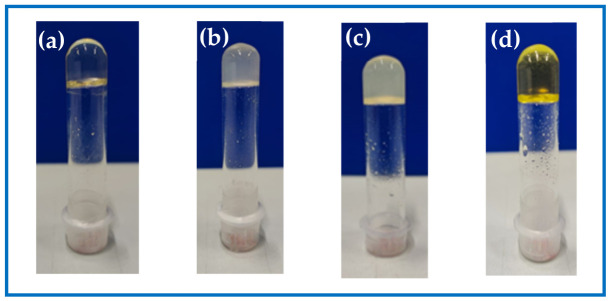
Selected examples of obtained gel phases through inverted test tubes experiments. (**a**) Clear gel (CG) with cyclohexane at 1.0% *w*/*w*; (**b**) opaque gel (OG) with *n*-hexane at 0.5% *w*/*w*; (**c**) opaque gel (OG) with THF at 2% *w*/*w;* and (**d**) clear gel (CG) with olive oil at 0.5% *w*/*w*.

The ability of AIPA–gallic acid to induce the gelation of organic solvents was probed using inverse test tubes experiments. The results are reported in Table 1 and indicate that AIPA–gallic acid gelled 11 of 20 different solvents tested, including all five different oils tested (i.e., paraffin, mineral and vegetable oils). Note that either clear transparent gels (CG) or opaque gels (OG) can form according to the nature of the solvent (see Figure 2 for selected examples), as already reported for AIPA supergelators [24].

Remarkably, AIPA–gallic acid also behaved as a supergelator with hexadecane, tetrachloromethane, ethyl acetate, *n*-hexane and olive oil (critical gelator concentration < 0.5% *w*/*w*).

To study the properties induced by AIPA–gallic acid in oil-based systems, the gelator was incorporated at concentrations of 2% and 4% by weight into three types of fats: mineral oil, vegetable oil, and cocoa butter. As reported in Table 1, at concentrations below 2% *w*/*w* in these systems, no gelation process occurs. Among the tested oils, these three types were selected for their specific structure and their differences in viscosity and rheological responses. All samples were homogeneous. They were prepared by adding AIPA–gallic acid to the oil at 120 °C under mechanical stirring for 2 h, a duration sufficient to ensure uniformity.

### 2.2. Morphology Studies

**Figure 3:**
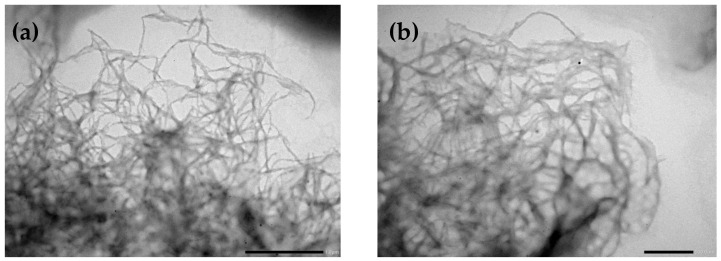
TEM images of the xerogel issued from the gelation of (**a**) cyclohexane (scale bar: 1.0 µm); (**b**) *n*-hexane (scale bar: 500 nm).

TEM images of the xerogels issued from the inverted test tubes experiments (Table 1) of the most volatile solvents revealed a texture resulting from the tight entanglement of flexible thin fibers several micrometers in length and from 2 to 5 nm of diameter, a texture that is often encountered in LMWGs [30]. Typical TEM micrographs are reported in Figure 3.

Notably, according to these images, the fibrils in both xerogels exhibited comparable dimensions. However, as shown in Figure 2, the gel formed in cyclohexane is transparent, whereas that in n-hexane appears opaque. Consequently, the difference in opacity is likely attributable to light-scattering effects arising from differences in the swelling property of the fibrils in the respective solvents.

### 2.3. Steady-State Sweep Test

The viscosity (η) of mineral oil at various shear rates across different temperatures (15 °C, 25 °C, 35 °C, 45 °C, 55 °C and 65 °C) and gelator concentrations (0%, 2%, and 4%) was evaluated at a shear rate of 10 s^−1^. This test reveals how the fluid behaves under constant shear conditions, which is crucial for applications where the fluid will be subjected to flow. 

A strain-controlled rheometer was used to characterize the rheological behavior of each sample. Steady shear measurements (flow curves) were conducted over a shear rate range of 0.1 to 100 s^−1^ and at temperatures between 15 and 65 °C.

Prior to these measurements, step shear rate tests were carried out at various shear rates to determine the equilibrium time required to reach steady-state conditions.

The viscosity (η) values are reported in Table 2 (See also Figures S1 and S2). The addition of additive has a pronounced effect on the viscosity of mineral and vegetable oils. At each temperature, increasing the gelator concentration from 0% to 4% resulted in a marked increase in viscosity. For example, at 25 °C, the viscosity rose from a baseline of 0.025 Pa·s (0% dosage) to 1.840 Pa·s (4% dosage). This suggests that the additive increases its resistance to flow, obviously enhancing the fluid structure, which is particularly beneficial in applications requiring thicker fluids. This is an important aspect in lubrication engineering where higher viscosity enhances the performance of lubricants under high temperature and pressure [31].

In addition, viscosity generally decreases with increasing temperature. This behavior is typical for most fluids, since higher temperatures provide more energy to the molecules, allowing them to overcome intermolecular forces more easily. Therefore, the interplay between temperature and additive dosage is critical. While higher temperatures reduce viscosity, the presence of additives can counteract this effect. For example, the viscosity is significantly high for the organogels at 4%, even at a temperature of 45 °C, indicating that the additives are effective in maintaining a higher viscosity at elevated temperatures. Consequently, at lower temperatures, the formulations with higher additive dosages tend to exhibit greater viscosity, showing how the additive enhances the oil’s ability to resist flow by creating a more structured network. This is particularly important for applications requiring stability and consistency in texture. However, as the temperature rises, the viscosity of the formulations with AIPA–gallic acid decreases in the same way as in oils without the supergelator; this suggests a stable/constant AIPA behavior across the entire temperature range, with no evident temperature-induced breakdown of the AIPA–gallic acid induced gelation.

A different phenomenon was observed in cocoa butter. For this material, which behaves more like a soft solid due to its very high viscosity, the viscosity value at 15 °C could not be determined because of limitations related to the experimental setup. At 25 °C, viscosity was measured at a shear rate of 10^−3^ s instead of 10 s^−1^; measurements at higher shear rates are not safe due to the solid-like nature of cocoa butter. The liquid state was explored at temperatures > 25 °C and characterized by a weak temperature dependence. Unlike mineral and vegetable oils, which demonstrated clear and stable viscosity increase with additive concentrations, the cocoa butter samples exhibited a lack of a similar effect overall. The data indicate that organogels did not form a cohesive network with the cocoa butter, which is essential for applications requiring structural integrity and stability. This indicates that the cocoa butter remained in a fluid state rather than transitioning into a gel-like consistency. Given these findings, it is clear that further analysis of cocoa butter is unwarranted. The inability of cocoa butter to increase its viscosity emphasizes the critical need for selecting suitable additives and concentrations to achieve the desired rheological properties. Further considerations coming for previous analysis [32] supports this indication. This situation also highlights the necessity for further research into alternative formulations or additives that could improve the gelation potential of cocoa butter.

Overall, the viscosity steady strain rate sweep test highlights the delicate balance between additive concentration and temperature in determining the rheological behavior of mineral and vegetable oils. Understanding these interactions is crucial for optimizing formulations for specific applications, ensuring that the desired texture and stability are maintained across varying thermal conditions. The data can guide manufacturers in optimizing formulations for desired performance in real-world applications, for example, in food product development (e.g., margarine, dressings, bakery items), where consistency and stability across various temperatures during processing, storage, and consumption are critical [33]. Viscosity provides an indication of gel formation; however, the gel network can be more thoroughly characterized using dynamic rheological experiments, as described in the next paragraph.

### 2.4. Frequency Sweep Tests

Frequency sweep tests investigate the material response to applied stress in oscillatory regimes, thus encompassing both its storage modulus (G′) and loss modulus (G″). Dynamic shear measurements were conducted over a frequency range of 0.1 to 15.9 Hz. Small-amplitude oscillatory tests were used to assess the linear viscoelastic properties of the materials, enabling definition, from a rheological point of view, of the gel-like behavior of the investigated systems. According to M.A. Rao [34], a gel can be defined as a “two-component system (e.g., gelling polymer and the solvent, water, or aqueous solution in foods) formed by a solid finely dispersed or dissolved in a liquid phase, exhibiting solid-like behavior under deformation”. A more proper definition [35] should underline that only when deformation is small enough, all gels (both physical and chemical ones) provide essentially the same gel mechanical spectrum, with G′>G″. According to these definitions, the systems studied in this work are gels: solid-like materials only when assessed under small deformations. G′ and G″ are slightly dependent on the frequency, and G′ is higher than G″ under small deformations.

**Figure 4:**
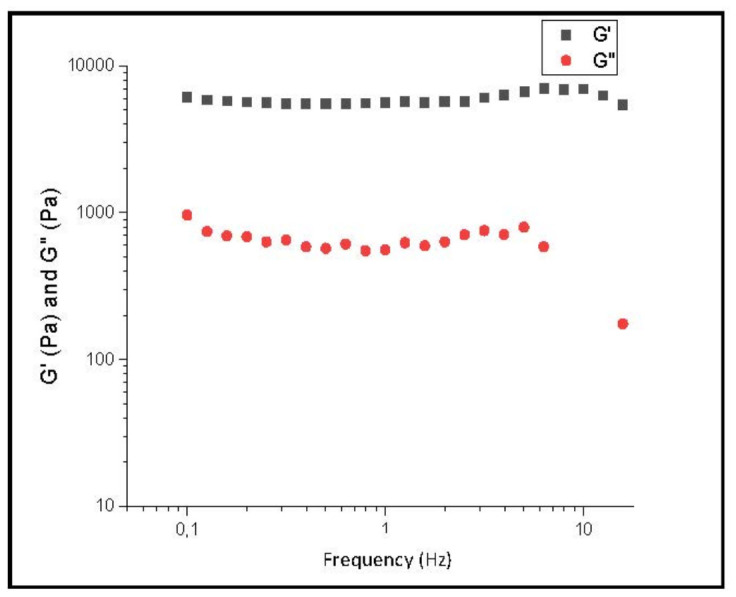
G′ and G′ versus frequency for gel with 4% *w*/*w* of AIPA–gallic acid in mineral oil at 15 °C.

Figure 4 shows a typical frequency sweep test where the elastic modulus (G′) and loss modulus (G″) are reported for the sample with mineral oil at 4% at 15 °C. The mechanical spectrum exhibits gel-like characteristics, with G′ significantly higher than G″ and both moduli are showing only weak frequency dependence, consistent with the rheological behavior of a gel.

The pure mineral oil behaved as a Newtonian fluid, where viscosity is independent on the shear rate and in the frequency sweep tests, G′ was not detectable and only G″ was observed. In contrast, the presence of our AIPA-derived additive made G′ and G″ dependent on the frequency. This is a typical change from a Newtonian fluid to a gel, induced by the presence of AIPA–gallic acid additive.

Therefore, AIPA-derived additive not only affects the viscosity from the macroscopic point of view but also changes the intermolecular structure to a real gel. For such a system, therefore, an analysis based on a weak gel model can be carried out [36]. This model derives from the theory of Bohlin [37] and Winter [38] and has been applied to several colloidal complex systems [31,39]. It should be noted that this approach is ultimately intended as a means of using a power-law constitutive equation to empirically describe the system’s rheological behavior, allowing easy comparison among various samples. In contrast, and following previously reported studies [37,38,39], theoretical arguments suggest that a cooperative arrangement of flow units connected by weak physical interactions cooperatively ensure the stability of the structure under gel shear. Thus, the weak-gel model links the microstructure of the material to its rheological properties. This regime is characterized by the flow, as shown in Equation (1):

(1) $$\left|G^{*}\left(\omega ,T,X\right)\right|=A\left(T,X\right)\omega^{\frac{1}{z(T,X)}}$$

In this equation, explicitly showing the dependencies on frequency (ω), temperature (T) and composition (X), G* is the mechanical modulus (G*^2^ = G′^2^ + G″^2^, encompassing the total energy that can be absorbed by the material under oscillatory stress, both elastically and inelastically), and A is a proper constant of the materials and depends on temperature and composition. The latter can be considered as the modulus at an angular frequency of 1 rad/s, so it may represent the specific characteristic of the interaction and therefore is called the strength of interaction. Given that G′ is significantly higher than G″, and G′ has values comparable to G*, it is strongly correlated with A. As a result, the trend of A closely follows that of G′. In contrast, z is the interacting rheological unit number, i.e., the number of neighboring molecules involved in the molecular motion under flowing process. This information is crucial for understanding how the additive interacts with mineral oil and how these interactions change with temperature.

The plots of ln|G*| vs. ln ω show lines with slope 1/z and intercept A. Both A and z are dependent on temperature and composition. In this representation, the frequency dependence of A and z is removed. Table 3 presents the strength of interaction (A) and the coordination number (z), as obtained by application of this model.

**Figure 5:**
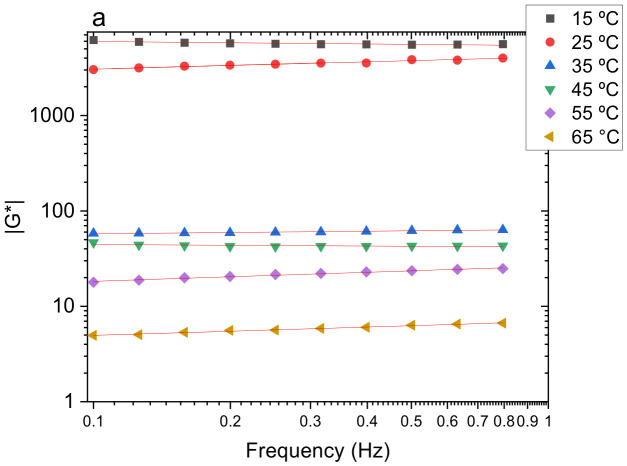

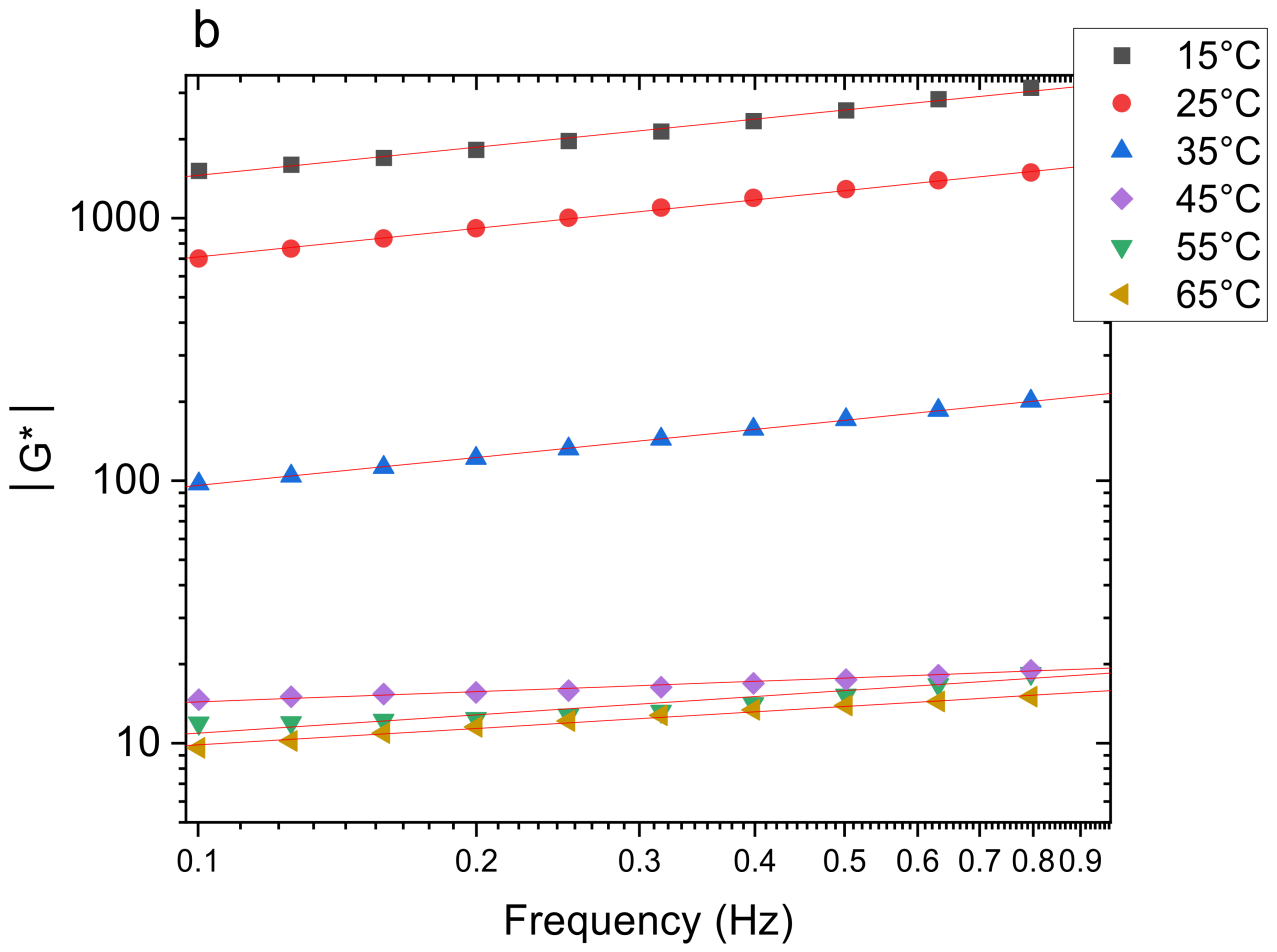
|G*| as a function of frequency for the mineral oil + 4% organogel additive sample (**a**) and the vegetable oil + 4% organogel additive sample (**b**). The continuous lines represent the linear fitting of the experimental data.

Figure 5 is provided as an example and shows the graphical fitting of G* behavior at 45 °C for the mineral + 4% organogel additive. Considering the complex modulus, Figure 5 clearly shows a decrease in the organogel’s mechanical strength and rigidity with increasing temperature and that the material behaves more like a fluid than a solid at higher temperatures. Significant variations in G* are observed above 25 °C, consistent with the changes in A values.

As shown in Table 3, A generally decreases with increasing temperature at gelator concentrations of 2 and 4 wt%. This trend suggests that as the temperature rises, crosslinking per junction is reduced, resulting in a looser gel network that is easier to disrupt. The z parameter also shows variability with temperature at both concentrations. At 2 wt%, z increases with increasing temperature, peaking at 45 °C. Above 45 °C, z decreases with increasing temperature. This data suggests that z is higher at lower temperatures, indicating that each additive molecule can form multiple connections with neighboring molecules (rheological units), creating a highly structured network. As the temperature increases, this network becomes increasingly disrupted by thermal energy and the gel becomes less structured, leading to a consistent decline in z. At 4%, z continuously decreases with increasing temperature, indicating that the gel becomes less structured over the entire temperature range.

In other words, the flow process generally involves neighboring molecules as described by Byran et al. [40]: “*For any one molecule to move, other surrounding molecules must first give way and move into vacant lattice sites or “holes” to create a space for the molecule to enter*”. In this situation, the thermal agitation disrupts some of the molecular interactions, fewer molecules remain close enough to attract each other strongly overall leading to a decrease in z. The interactions themselves may also change, but together, these effects self-consistently lead to more fluid-like behavior at elevated temperatures, affecting the overall performance of mineral oil. Interestingly, a tendency to peak at around 45 °C, is maintained. This suggests that at lower concentrations, temperature may initially enhance coordination by facilitating the formation of connections between fewer available molecules before thermal agitation weakens these interactions. This effect is more evident for lower additive content, where a progressive increase in z value with the increasing temperature is observed. Concentration and temperature clearly have an effect on the supramolecular structure. However, at temperatures high enough, the common trend gives a general lower coordination number (z) than at lower temperatures, thus reflecting the reduced molecular organization and supporting the parallel observed decline in interaction strength (A).

The strength of interaction and the coordination number for the organogel at 4% are higher than those at the 2%. This is consistent with the viscosity measured under constant shear. This increase indicates that the additive molecules form more robust and numerous connections with mineral oil, enhancing the overall stability and performance of the formulation. Stronger interactions at 4% may lead to a more robust gel-like structure, which is beneficial for applications requiring enhanced viscosity and stability. The increasing z value with the additive content also reflects its capability in forming an interconnected structure: each molecule (rheological units) [30] can form additional connections with neighboring molecules, leading to a denser and more cohesive network. This is particularly evident at lower temperatures and higher AIPA–gallic acid content, where the coordination number is maximized, indicating a well-structured gel-like formation that is less likely to break down under stress.

The role of AIPA–gallic acid content is therefore crucial: at 4%, the coordination number at the highest temperature is still higher than at 2% at the lowest temperature. This example clearly shows that the enhanced network formed at higher concentrations provides better thermal stability, allowing the material to retain its structural integrity and performance characteristics even at elevated temperatures. This is in accordance with the behavior shown by steady-state viscosity. Maintaining viscosity and structural stability under extreme heat is essential in high-performance engines, as lubricants must perform reliably under high temperatures and shear conditions [41]. This indicates that the 4% formulation is likely to perform better in applications where temperature fluctuations are common, maintaining its high viscosity and stability more effectively than the 2% formulation.

Regarding the vegetable oil, the parameters derived through the weak-gel model are reported in Table 4.

In vegetable oil, the temperature effect is similar to that observed for mineral oil:

1. The A value clearly decreases with increasing T, indicating that intermolecular forces within the gel network weaken with higher thermal energy. z generally decreases with increasing temperature, further indicating that the gel’s ability to maintain its network structure diminishes as the temperature rises.
2. Increasing the gelator concentration results in higher A values, indicating a more robust network formation that enhances the stability and performance of the oil. z demonstrates a greater extent of connectivity among the molecules, reflecting a more interconnected structure that contributes to the overall viscosity and texture of the formulation.

In contrast to mineral oil, the temperature dependence of z for the gels with vegetable oil is flat (with no peaking tendency). This confirms the importance of considering temperature and AIPA–gallic acid content effects but underscores the specificity of the matrix.

Generally, the trend against temperature demonstrates that at lower temperatures, z is generally higher, reflecting stronger molecular interactions, a more robust network structure, and a greater number of rheological units (structural units contributing to the material’s flow behavior). For the 2% dosage, the tendency to z to peak at intermediate temperatures holds, with z showing an initial increase, followed by a sharp decrease as temperature rises. This indicates a significant loss of coordination and a reduction in the number of effective rheological units due to thermal agitation. For temperatures higher than 45 °C, vegetable oil is a Newtonian fluid. For the 4% dosage, z remains relatively stable, with smaller fluctuations, suggesting a more resilient network to temperature changes, likely due to the higher concentration stabilizing interactions and preserving the integrity of rheological units. Overall, the data illustrates that higher additive concentrations enhance thermal stability but may experience clustering or saturation effects, reducing the effective number of rheological units slightly compared to lower concentrations.

In this work, cocoa butter represents a peculiar case. A different phenomenon was observed for cocoa butter. For this material, AIPA–gallic acid addition did not trigger gelation. AIPA-containing cocoa butter is a Newtonian fluid at any temperature. This confirms what was found by steady-state sweep tests, indicating that our AIPA-derived gelator did not form a gel with cocoa butter. Cocoa butter remains in a Newtonian fluid state rather than transitioning into a gel-like consistency, confirming the need for further research on alternative formulations or additives to improve the gelation of cocoa butter.

To understand the molecular mechanism of gelation when AIPA–gallic acid is added to an oil or cocoa butter, one must consider the simultaneous presence of carboxylic acid, amide and aromatic groups. It is known that the simultaneous presence of acidic and basic groups causes strong intermolecular interactions and the formation of an extended network, as observed in some model systems [42]. Specifically, the gelation general mechanism is expected to be based on the formation of cyclic hydrogen-bonded hexamers via the carboxylic acid groups of the isophthalic acids [24]. These hexamers then stack into elementary fibers stabilized by hydrogen bonding of the amide linker groups and π–π stacking interactions between the aromatic isophthalic acid rings. This supramolecular organization leads to the formation of a three-dimensional network holding up the whole structure, including the solvent, and resulting in gelation. This is quite a complex mechanism involving a hierarchical self-assembly process, where the length of alkyl chain, which directly interacts with the oil, can also play an important role, influencing the efficiency and stability of gelation.

To rationalize the differences among mineral oil, vegetable oil and cocoa butter, their chemistry needs to be considered. The mineral oil (Nypar 300, classified as a heavy paraffinic compound) can offer good compatibility with the alkyl chains of AIPA. However, here, another mechanism is also present, specifically, the mineral oil tendency to align its chains in parallel to maximize van der Waals interactions. This tendency is obviously in competition with the tendency to establish interactions with the alkyl chains of the rather bulky AIPA molecules. Increasing temperature has a disrupting effect, but this can be exerted in different ways (i.e., at different extent) for oil–oil and oil–AIPA interactions. As a result, a maximum in a structural rheological parameter (in particular, the coordination number, z) can hold and was indeed observed for mineral oil around 45 °C. This peaking was not seen for vegetable oil, suggesting that in this case, oil–oil interactions do not counterbalance, or are not opposed to, oil–AIPA interactions. As a consequence, no maximum in z parameter was observed in vegetable oil. Vegetable oil has a different chemical nature: it is not paraffinic but rather fatty, consisting mainly of esters, containing around 80% mono- and poly-unsaturated fatty acids, for which the alkyl–alkyl competition found in paraffinic oils is much less pronounced. At the other extreme, cocoa butter is a solid fat with a different structure (see ref. [32] for details), which further hinders the gelation mechanism. Therefore, further experiments are still needed to find the best formulation or gelator.

## 3. Conclusions

A novel LMWG AIPA–gallic acid was successfully synthesized and suitably functionalized to induce gelation in various kinds of oil matrices. Notably, even a concentration as low as 2 wt% emerged as effective in inducing gelation in mineral oil and vegetable oil. Cocoa butter, in contrast, is solid at 15 °C, so the flow curve that can be obtained at this temperature should show high values not comparable with the other liquid oils. In this material, however, at higher temperatures, gelation was found to be hindered, most probably due to the solid-like and complex crystalline structure.

Rheological analysis, carried out by the evaluation of storage and loss moduli as a function of shear rate, frequency, and temperature in the viscoelastic region, consistently showed that the gel-forming mechanism induced by AIPA–gallic acid causes an increase in the coordination number during the flow process. This, in turn, suggests the formation of a well-structured gel network within the oil matrix. This observation confirms that the organogel effectively interacts with the oil molecules. Incidentally, no slippage phenomenon was observed during the rheological measurements, and no time-dependent behavior (thixotropy) was observed.

Interestingly, the gelation effect was present across the entire studied temperature range (15–65 °C), proving the versatility of AIPA–gallic acid to gel various kinds of oils over a wide range of temperatures and showing that AIPA–gallic acid preserves the oils from the breakdown of viscosity usually observed upon temperature increase.

In particular, the potential of AIPA–gallic acid to enhance the rheological properties of mineral and vegetable oils has been shown. However, the structural differences between these two oils, as well as cocoa butter’s unique challenges due to its solid fat content and complex crystalline structure hindering gelation, necessitate further exploration. Future research should focus on identifying new compounds that could improve gelation potential across an even broader list of oil types and temperatures, for both technological and culinary applications.

## 4. Materials and Methods

### 4.1. Materials

AIPA–gallic acid was synthesized by suitably modifying reported procedures [24,43], starting from commercially available methyl gallate and dimethyl 5-aminoisophthalate. In the first step, methyl gallate was functionalized by Williamson etherification to introduce the three octadecyl alkyloxy chains. Saponification of the methyl ester group and subsequent conversion of the benzoic acid group to benzoyl chloride was then achieved. The thus obtained alkylated galloyl chloride was reacted with dimethyl-5-aminoisophthalate, and the condensed adduct was finally saponified with potassium hydroxide to yield the AIPA–gallic acid. Details of the synthetic procedure in each step are reported in ESI. 

The Nypar 300 was a free sample kindly offered by the NYNAS enterprise (Hammarbybacken 27, Stockholm, Sweden). It is a mineral oil and classified as a heavy paraffinic hydrotreated petroleum distillate. Further details are available in the literature [20].

The vegetable oil, specifically sunflower oil, was produced by Olearia Desantis S.p.a. (Bitonto, BA, Italy). It was chosen for its straightforward availability, consistent composition, and suitability for the experimental requirements, making it preferable to other vegetable oils. Further details are available in the literature [8]. It was purchased from a local supermarket in Italy.

The cocoa butter was a commercial sample whose characterization has been previously reported in the literature [27].

### 4.2. Gelation

The gelation ability of AIPA–gallic acid in various solvents and oils was evaluated using inverted test tube experiments. To this end, the appropriate amount of AIPA–gallic acid was dissolved in 0.5 mL of solvent inside a sealed 4 mL vial (15 mm in diameter, 45 mm in height). The sealed vials were then immersed in an oil bath at 120 °C for 10 min and subsequently allowed to cool to room temperature. After standing for 30 min in ambient conditions, the vials were inverted to assess the viscosity and phase behavior of the resulting material.

### 4.3. Rheology

Rheological measurements were performed using a shear strain-controlled rheometer, Rheometrics RFS III, equipped with either a cone–plate geometry (diameter 50 mm, angle 0.0391 rad, gap 0.0483 mm) or a standard double-gap Couette geometry (bob length of 39 mm, gaps of 1 mm, external radius of 34 mm), depending on the viscosity of the samples. Temperature control was achieved using either a Peltier system (±0.1 °C) or a water circulator (±0.2 °C). All measurements were performed in triplicate.

### 4.4. Transmission Electron Microscopy

Morphologic images of the xerogels were taken by Transmission Electron Microscopy (TEM) with a Jeol Microscope (JEM-1400 Plus 120 kV, Tokyo, Japan). The samples for (TEM) were prepared by deposition of a drop of the gel solutions on 300 mesh copper grids. The xerogels were observed at an operating voltage of 80 kV after complete evaporation of the solvent in air.

## Figures and Tables

**Table 1:** Appearances of AIPA–gallic acid in different liquids according to its concentration.

Liquid	Concentration of AIPA–Gallic Acid (*w*/*w*)					
	0.5%	1%	1.5%	2%	2.5%	5%
Water	I					
Methanol	I					
Ethanol	S	S	OG			
Acetonitrile	I					
Acetone	I					
Dimethyl Sulfoxide	S	S	S	S	S	S
Dodecane	I					
Hexadecane	OG					
Chloroform	S	S	S	S	S	OG
Tetrachloromethane	CG					
Ethyl Acetate	OG					
Tetrahydrofuran	S	S	S	OG		
Dioxane	I					
Glycol Ethylene	I					
Toluene	S	S	CG			
Xylene	S	S	S	S	S	S
Mesitylene	S	S	S	S	S	CG
Cyclohexane	S	CG				
*n*-Hexane	OG					
Aniline	S	S	CG			
Paraffin Oil	CG					
Olive Oil	CG					
Engine Oil ^(a)^	S	CG				
Mineral Oil ^(b)^	S	S	S	CG		
Vegetable Oil ^(c)^	S	S	S	CG		

^(a)^ Waste triglycerides by biodiesel process ILSAP; ^(b)^ Nypar 300; ^(c)^ sunflower oil. S: solution; I: insoluble; CG: clear gel; OG, opaque gel.

**Table 2:** Viscosities (η) at a shear rate of 10 s^−1^ for different temperatures and gelator concentrations (0, 2 and 4%). Data marked with asterisks refer to a shear rate of 10^−3^ s; higher shear rate measurements were not safe due to the solid nature of the cocoa butter.

T (°C)	Mineral Oil (Pa·s)			Vegetable Oil (Pa·s)			Cocoa Butter (Pa·s)		
	0%	2%	4%	0%	2%	4%	0%	2%	4%
15	0.025	1.32	6.05	1.04	2.2	204			
25	0.035	1.38	1.84	0.025	1.85	54.38	2.2 × 10^6^ *	2.2 × 10^6^ *	2.2 × 10^6^ *
35	0.021	0.572	1.11	0.020	1.13	3.90	0.04	0.067	0.087
45	0.004	0.160	0.494	0.0127	0.11	0.85	0.026	0.042	0.065
55	0.003	0.0642	0.1647	0.0098	0.085	0.23	0.021	0.036	0.046
65	0.002	0.0442	0.0819	0.0085	0.052	0.077	0.016	0.024	0.030

**Table 3:** Strength of interaction (**A**) and coordination number (**z**) for organogels of AIPA–gallic acid with mineral oil (2 and 4%).

Temperature	AIPA–Gallic Acid Content 2 wt%		AIPA–Gallic Acid Content 4 wt%	
	A (±0.1, Pa·s^−1/Z^)	z (±0.1)	A (±0.1, Pa·s^−1/Z^)	z (±0.1)
15 °C	128.0	4.3	5927.1	37.3
25 °C	120.0	5.8	3180.7	23.1
35 °C	112.0	8.0	66.1	13.1
45 °C	41.0	17.4	45.5	21.2
55 °C	3.0	4.6	25.6	7.2
65 °C	0.7	2.1	7.1	6.0

**Table 4:** Strength of interaction (**A**) and coordination number (**z**) for vegetable oil.

Temperature	AIPA–Gallic Acid Content 2 wt%		AIPA–Gallic Acid Content 4 wt%	
	A (±0.1, Pa·s^−1/Z^)	z ± 0.1	A (±0.1, Pa·s^−1/Z^)	z ± 0.1
15 °C	216.4	5.2	3745.6	3.1
25 °C	93.8	7.5	1439.4	3.3
35 °C	40.5	4.9	222.3	3.1
45 °C	7.1	3.2	28.1	2.7
55 °C	---	---	24.9	3.2
65 °C	---	---	17.8	2.6

## Data Availability

The original contributions presented in this study are included in the article/Supplementary Material. Further inquiries can be directed to the corresponding authors.

## Supplementary Materials

The following supporting information can be downloaded at https://www.mdpi.com/article/10.3390/gels12060482/s1. Scheme S1: Synthetic pathway to AIPA-Gallic acid. Reagent and conditions: (i) 1 Bromo-octadecane, KI (cat.), cyclohexanone; (ii) KOH/MeOH, 4 h reflux (iii) HCl (1 M); (iv) SOCl_2_, CCl_4_, 3 h, reflux; (v) Et_3_N cat., CCl_4_, r.t., 24 h; (vi) KOH/MeOH, 4 h reflux; (vii) HCl (1 M). Figure S1. Flow curves of the Vegetable oil at various temperatures: without additive (a), with 2% *w*/*w* AIPA–gallic acid (b), and with 4% *w*/*w* AIPA–gallic acid (c). Figure S2. Flow curves of the Mineral oil at various temperatures: without additive (a), with 2% *w*/*w* AIPA–gallic acid (b), and with 4% *w*/*w* AIPA–gallic acid (c).

## References

[1] Barnes H.A. (2000). A Handbook of Elementary Rheology.

[2] Foster R., Williamson C.S., Lunn J. (2009). Culinary Oils and Their Health Effects. Nutr. Bull..

[3] Martins A.J., Vicente A.A., Cunha R.L., Cerqueira M.A. (2018). Edible Oleogels: An Opportunity for Fat Replacement in Foods. Food Funct..

[4] Silva R.C.d., Ferdaus M.J., Foguel A., da Silva T.L.T. (2023). Oleogels as a Fat Substitute in Food: A Current Review. Gels.

[5] Wang J., Zhang L., Tang H., Liu Y., Wang Y., Wang X., Gao M. (2025). Novel Biodegradable Extinguishing Gel: Preparation, Fire-Extinguishing Performance and Mechanism Study. Colloids Surf. A Physicochem. Eng. Asp..

[6] Terech P., Weiss R.G. (1997). Low Molecular Mass Gelators of Organic Liquids and the Properties of Their Gels. Chem. Rev..

[7] Guenet J.-M. (2016). Organogels.

[8] Draper E.R., Adams D.J. (2017). Low-Molecular-Weight Gels: The State of the Art. Chem.

[9] Weiss R.G., Terech P. (2006). Molecular Gels.

[10] Weiss R.G. (2018). Molecular Gels.

[11] Chivers P.R.A., Smith D.K. (2019). Shaping and Structuring Supramolecular Gels. Nat. Rev. Mater..

[12] Yu X., Chen L., Zhang M., Yi T. (2014). Low-Molecular-Mass Gels Responding to Ultrasound and Mechanical Stress: Towards Self-Healing Materials. Chem. Soc. Rev..

[13] Smith D.K. (2024). Supramolecular Gels—A Panorama of Low-Molecular-Weight Gelators from Ancient Origins to next-Generation Technologies. Soft Matter.

[14] Suzuki M., Hayakawa Y., Hanabusa K. (2015). Thixotropic Supramolecular Gel Based on L-Lysine Derivatives. Gels.

[15] López-Pedrouso M., Lorenzo J.M., Gullón B., Campagnol P.C.B., Franco D. (2021). Novel Strategy for Developing Healthy Meat Products Replacing Saturated Fat with Oleogels. Curr. Opin. Food Sci..

[16] Nikam A.N., Roy A., Raychaudhuri R., Navti P.D., Soman S., Kulkarni S., Shirur K.S., Pandey A., Mutalik S. (2024). Organogels: “GelVolution” in Topical Drug Delivery—Present and Beyond. Curr. Pharm. Des..

[17] Makeiff D.A., Cho J.-Y., Godbert N., Smith B., Azyat K., Wagner A., Kulka M., Carlini R. (2021). Supramolecular gels from alkylated benzimidazolone derivatives. J. Mol. Liq..

[18] Martinez R.M., Rosado C., Velasco M.V.R.R., Lannes S.C.S.S., Baby A.R. (2019). Main Features and Applications of Organogels in Cosmetics. Int. J. Cosmet. Sci..

[19] Vintiloiu A., Leroux J.C. (2008). Organogels and Their Use in Drug Delivery—A Review. J. Control. Release.

[20] Pinto T.C., Martins A.J., Pastrana L., Pereira M.C., Cerqueira M.A. (2021). Oleogel-Based Systems for the Delivery of Bioactive Compounds in Foods. Gels.

[21] Fiorica C., Biscari G., Palumbo F.S., Pitarresi G., Martorana A., Giammona G. (2021). Physicochemical and Rheological Characterization of Different Low Molecular Weight Gellan Gum Products and Derived Ionotropic Crosslinked Hydrogels. Gels.

[22] Liu X.Y., Li J. (2013). Soft Fibrillar Materials.

[23] Potluri V.K., Hamilton A.D. (2002). Isophthalic Acid-Derived Organogelators. J. Supramol. Chem..

[24] Makeiff D.A., Cho J.-Y., Smith B., Carlini R., Godbert N. (2022). Self-Assembly of Alkylamido Isophthalic Acids toward the Design of a Supergelator: Phase-Selective Gelation and Dye Adsorption. Gels.

[25] Patel A.R., Dewettinck K. (2016). Edible Oil Structuring: An Overview and Recent Updates. Food Funct..

[26] Zhang F., Zhang Q., Zhou Y., Zhou Z., Luo C., Wang Y., Yao B., Ji X. (2021). Comparative Study of the Micro-Rheological Properties and Microstructure of Edible Oil Gels Prepared by Amino Acid Gelator. Colloids Surf. A Physicochem. Eng. Asp..

[27] Loke Y.H., Phang H.C., Mohamad N., Kee P.E., Chew Y.-L.L., Lee S.-K.K., Goh C.F., Yeo C.I., Liew K. (2024). Bin Cocoa Butter: Evolution from Natural Food Ingredient to Pharmaceutical Excipient and Drug Delivery System. Planta Medica.

[28] Azyat K., Makeiff D., Smith B., Wiebe M., Launspach S., Wagner A., Kulka M., Godbert N. (2023). The Effect of Branched Alkyl Chain Length on the Properties of Supramolecular Organogels from Mono-N-Alkylated Primary Oxalamides. Gels.

[29] Caputo P., Aiello I., Caligiuri R., Giorno E., Abe A.A., Oliviero Rossi C., Godbert N. (2022). Polyalkylated Gallic Esters and Acids, High Performant Warm Mix Asphalt and Adhesion Promoters for Bitumen. Int. J. Adhes. Adhes..

[30] Tang Y.T., Dou X.Q., Ji Z.A., Li P., Zhu S.M., Gu J.J., Feng C.L., Zhang D. (2013). C2-Symmetric Cyclohexane-Based Hydrogels: A Rational Designed LMWG and Its Application in Dye Scavenging. J. Mol. Liq..

[31] Coppola L., Gentile L., Nicotera I., Rossi C.O., Ranieri G.A. (2010). Evidence of Formation of Ammonium Perfluorononanoate/^2^H_2_O Multilamellar Vesicles: Morphological Analysis by Rheology and Rheo-^2^H NMR Experiments. Langmuir.

[32] Colella M.F., Marino N., Oliviero Rossi C., Seta L., Caputo P., De Luca G. (2023). Triacylglycerol Composition and Chemical-Physical Properties of Cocoa Butter and Its Derivatives: NMR, DSC, X-Ray, Rheological Investigation. Int. J. Mol. Sci..

[33] Fallahasgari M., Barzegar F., Abolghasem D., Nayebzadeh K. (2023). An Overview Focusing on Modification of Margarine Rheological and Textural Properties for Improving Physical Quality. Eur. Food Res. Technol..

[34] Rao M.A. (2010). Rheology of Fluid and Semisolid Foods Principles and Applications.

[35] Ross-Murphy S.B. (1995). Rheological Characterisation of Gels. J. Texture Stud..

[36] Gabriele D., De Cindio B., D’Antona P. (2001). A Weak Gel Model for Foods. Rheol. Acta.

[37] Bohlin L. (1980). A Theory of Flow as a Cooperative Phenomenon. J. Colloid Interface Sci..

[38] Winter H.H. (1987). Can the Gel Point of a Cross-linking Polymer Be Detected by the G′–G″ Crossover?. Polym. Eng. Sci..

[39] Oliviero C., Coppola L., La Mesa C., Ranieri G.A., Terenzi M. (2002). Gemini Surfactant–Water Mixtures: Some Physical–Chemical Properties. Colloids Surf. A Physicochem. Eng. Asp..

[40] Bryan J., Kantzas A., Bellehumeur C. (2005). Oil-Viscosity Predictions From Low-Field NMR Measurements. SPE Reserv. Eval. Eng..

[41] Martini A., Ramasamy U.S., Len M. (2018). Review of Viscosity Modifier Lubricant Additives. Tribol. Lett..

[42] Calandra P., Mandanici A., Liveri V.T. (2013). Self-Assembly in Surfactant-Based Mixtures Driven by Acid-Base Reactions: Bis(2-Ethylhexyl) Phosphoric Acid-n-Octylamine Systems. RSC Adv..

[43] Zhambolova A., Vocaturo A.L., Tileuberdi Y., Ongarbayev Y., Caputo P., Aiello I., Oliviero Rossi C., Godbert N. (2020). Functionalization and Modification of Bitumen by Silica Nanoparticles. Appl. Sci..

