# Supplementary material for: Effects of a Low-Molecular-Weight Gelator in Vegetable, Mineral Oil and Cocoa Butter: A Comparative Rheological Study
Source: Gels. 2026 Jun 1;12(6):482. doi: 10.3390/gels12060482 (PMC13298344; doi:10.3390/gels12060482)
Supplement: Supplementary file 1 [file gels-12-00482-s001.zip › gels-4248412-supplementary.pdf]

# SUPPORTING INFORMATION

# Effects of a Low-Molecular-Weight Gelator in Vegetable, Mineral Oil and Cocoa Butter: A Comparative Rheological Study

**Emmanuel Anegbe [1], Cesare Oliviero Rossi [1], Iolinda Aiello [2,3,4,*], Nicolas Godbert [2,3,*], Eugenia Giorno [2], Darren A. Makeiff [5], Pietro Calandra [6,*] and Paolino Caputo [1]**

[1] *Dipartimento di Chimica e Tecnologie Chimiche, Università della Calabria, UdR INSTM della Calabria Via P. Bucci, Cubo 14/D, 87036 Rende (CS), Italy*

[2] *MAT-InLAB, LASCAMM CR-INSTM, Unità INSTM della Calabria, Dipartimento di Chimica e Tecnologie Chimiche, Università della Calabria, Via P. Bucci 14/C, 87036 Rende (CS), Italy*

[3] *LPM-Laboratorio Preparazione Materiali, Star-Lab, Università della Calabria, 87036 (CS), Italy*

[4] *CNR NANOTEC, UOS Rende c/o Dipartimento di Fisica, Università della Calabria, via P. Bucci 33/C, 87036 Rende (CS), Italy*

[5] *Quantum and Nanotechnologies Research Centre, National Research Council*

*Canada, 11421 Saskatchewan Drive, Edmonton, AB, T6G 2M9, Canada*

[6] *CNR-ISMN, National Research Council, Institute of Nanostructured Materials, Strada Provinciale 35 D n.9, 00010 Montelibretti, RM, Italy*

* *Corresponding Authors: iolinda.aiello@unical.it, nicolas.godbert@unical.it, pietro.calandra@cnr.it*

## Synthesis of AIPA-Gallic acid

### Reagents and conditions

All commercially available reagents and solvents were purchased from Merck or Alfa Aesar and used without further purification.

IR spectra (KBr pellets) were recorded on a Perkin-Elmer Spectrum 100 FT-IR spectrometer. $^{1}$H-NMR spectra were recorded on a Bruker Avance 500 MHz spectrometer and in deuterated chloroform ($CDCl_3$ or DMSO-d6) with TMS as internal standard. Elemental analyses were performed with a Perkin-Elmer 2400 analyser CHNS/O. Melting points were determined with a Leica DMLP polarizing microscope equipped a Leica DFC280 camera and CalCTec (Italy) heating stage.

AIPA-Gallic acid was synthesised from Gallic acid following a multistep synthetic pathway summarised in Scheme S1.

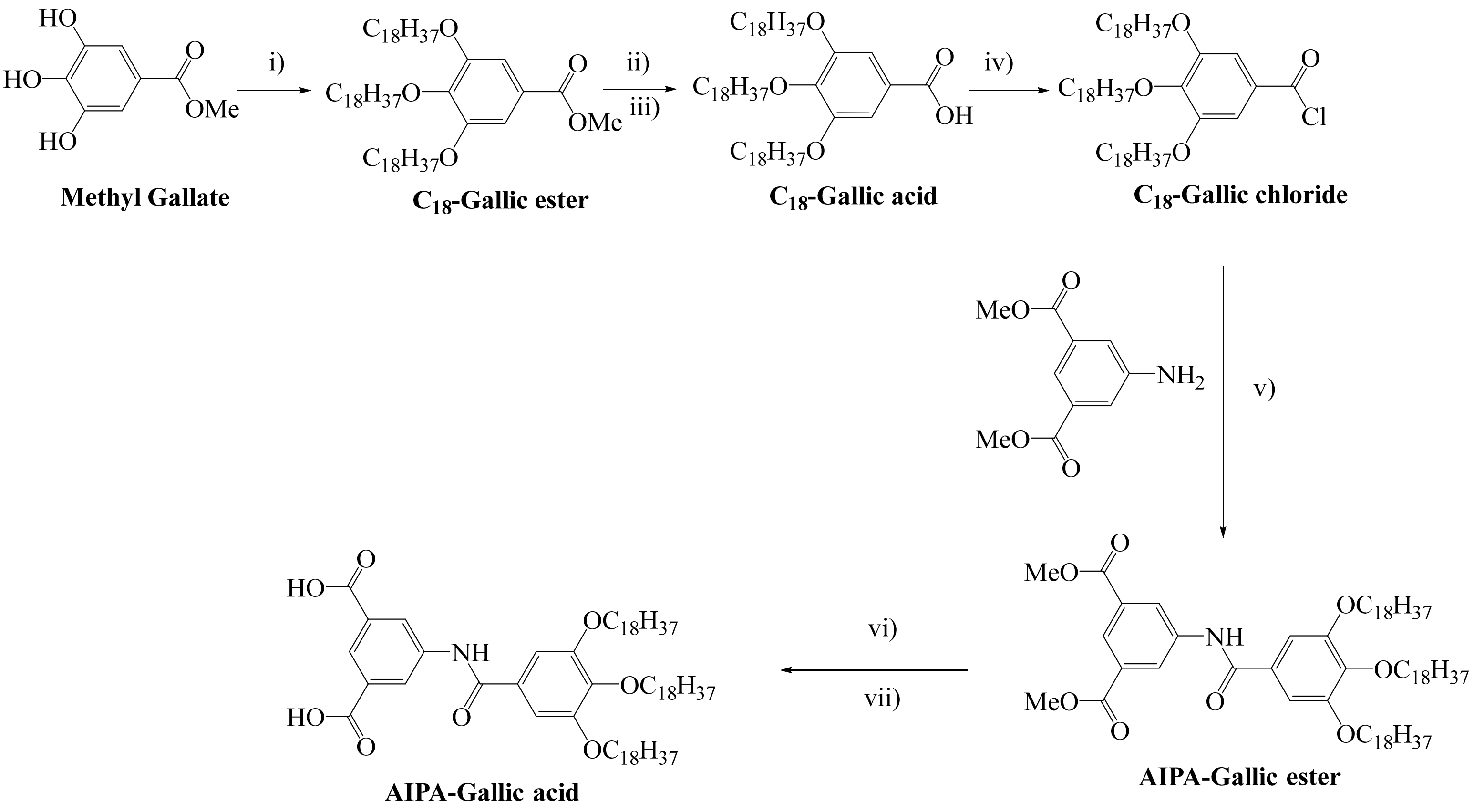


Scheme S1: Synthetic pathway to AIPA-Gallic acid. Reagent and conditions: i) 1 Bromo-octadecane, KI (cat.), cyclohexanone; ii) KOH/MeOH, 4h reflux iii) HCl (1M); iv) $SOCl_2$, $CCl_4$, 3h, reflux; v) $Et_3N$ cat., $CCl_4$, r.t., 24h; vi) KOH/MeOH, 4h reflux; vii) HCl (1M)

A) Synthesis of **$C_{18}$-Gallic ester**

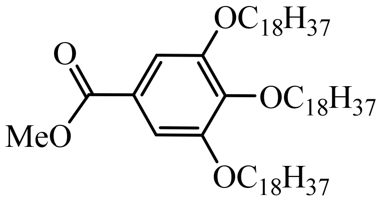

**$C_{18}$-Gallic ester**

Methyl gallate (3.16 g, 1,71.$10^{-2}$ mol), 1-bromooctadecane (18.82 g, 5.64 $\cdot 10^{-2}$ mol), $K_2CO_3$ (14.01 g, 1.02$\cdot 10^{-1}$ mol) and a catalytic amount of KI were stirred in cyclohexanone and refluxed for 48h. The resulting mixture was cooled down to room temperature, filtered and the solvent was evaporated under reduced pressure with addition of water for azeotropic distillation. The resulting oil was dissolved in ethanol and kept in the fridge for 24 hours upon which period of time a white precipitate was formed. The white solid was filtered, washed with water and cold ethanol.

White solid, 12.6 g, yield 78%, m.p.(°C): 68 °C. FT-IR (KBr) ν ($cm^{-1}$): 2918, 2848 ($CH_x$), 1716 (C=O), 1587, 1434 (C=C). $^1$H-NMR (500 Mhz, $CDCl_3$) δ (ppm): 7.24 (s, 2H), 4.02 (t, 6H), 3.98 (s, 3H), 1.88-1.71 (m, 6H), 1.47-1.42 (m, 6H), 1.24-1.19 (m, 84H), 0.90-0.86 (m, 9H); anal. ($C_{62}H_{116}O_5$): calcd. C: 79.09, H: 12.42, O: 8.50%; found C: 78.91, H: 12.37, O: 8.76%.

B) Synthesis of **$C_{18}$-Gallic acid**

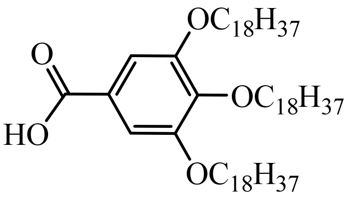

**$C_{18}$-Gallic acid**

**$C_{18}$-Gallic ester** (6.00 g, 6,3.$10^{-3}$mol) was suspended in a potassium hydroxide (0.71 g) solution in ethanol (140 mL). The resulting mixture was refluxed for 24 hours. After cooling down to room temperature, acidification of the mixture was performed by adding a solution of hydrochloric acid (1M) until pH = 1. The resulting white precipitate was filtered and washed with water (2x100 mL) and cold ethanol (2x100 mL). White solid, 4.968 g, yield 85 %, m.p. (°C): 75 °C. FT-IR (KBr) ν ($cm^{-1}$): 3500-3000 (OH), 2917, 2849 ($CH_x$), 1686.42 (C=O), 1587.63, 1469.59, 1429 (C=C). $^1$H-NMR (500 Mhz, $CDCl_3$) δ (ppm): 7.26 (s, 2H), 4.0-3.87 (m, 6H), 1.82-1.70 (m, 6H), 1.48 (m, 6H), 1.22 (m, 84H), 0.90 (m, 9H); anal. ($C_{61}H_{114}O_5$): calcd. C: 78.99, H: 12.39, O: 8.62%; found C: 79.18, H: 12.25, O: 8.78%.

C) Synthesis of **$C_{18}$-Gallic chloride**

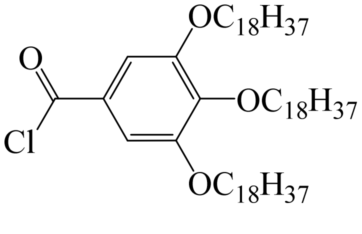

**$C_{18}$-Gallic chloride**

**$C_{18}$-Gallic ester** (2,5 g, 269 mmol) was dissolved in 30 ml of tetrachloromethane ($CCl_4$) at room temperature. Thionyl chloride (0.40 mL, 539 mmol) was added and the resulting mixture was refluxed for 2 hours. After cooling down to room temperature, the solvent was evaporated under reduced pressure. Successive and consecutive additions of $CCl_4$ were added (3 times) and each time solvent was removed under reduced pressure. The resulting obtained crude waxy white solid was directly used without any further purification.

Waxy white solid, 7.90 g. $^1$H-NMR (500 Mhz, $CDCl_3$): 7.32 (s, 2H), 4.10-3.98 (m, 6H), 1.9-1.7 (m, 6H), 1.5-1.2 (m, 84H), 0.9-0.8 (m, 9H). Note that due to its facile hydrolysis, no further characterization was performed.

D) Synthesis of **AIPA-Gallic ester**

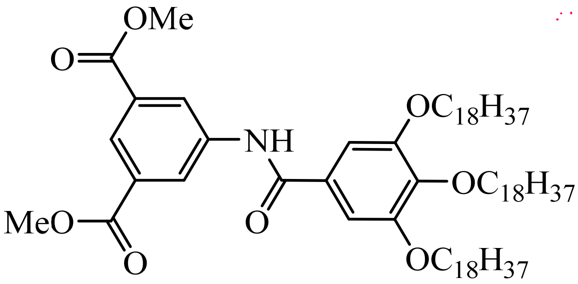

**AIPA-Gallic ester**

**$C_{18}$-Gallic chloride** (2.55 g, 269 mmol) and dimethyl 5-aminoisophthalate (0.56 g, 269 mmol) were dissolved in $CCl_4$ (30 mL). Triethylamine (0.74 mL, 539 mmol) was then added to the mixture and the reaction was stirred at 100°C for 3 hours, and maintained at room temperature for further 24 hours. The resulting crude product was extracted by adding 250 mL of dichloromethane and 150 mL di distilled water. The organic phase was collected, dried over sodium sulphate, and the solvent was evaporated under reduced pressure. The crude oil is then triturated in methanol until precipitation of a beige solid.

Beige solid, 2.51g, Yield 99% (2.51 g), m.p.: 70° C, FT-IR (KBr) ν ($cm^{-1}$): 3600-3200, 2919, 2849 ($CH_x$), 1732 (C=O), 1649 (C=O), 1584, 1505, 1470 (C=C), 1242, 1123, 854, 753, 719; $^1$H-NMR (500 MHz, DMSO) δ (ppm): 8.53 (s, 2H), 8.46 (s, 1H), 8.0 (br s, 1H, NH), 7.07 (s, 2H), 4.1-3.9 (m, 6H), 3.96 (s, 6H), 1.9-1.7 (m, 6H), 1.5-1.4 (m, 6H), 1.3-1.1 (m, 84H), 0.9-0.8 (m, 9H); anal. ($C_{71}H_{123}NO_8$): calcd. C: 76.23, H: 11.08, N: 1.25%; found C: 76.02, H: 11.23, N: 1.37%.

E) Synthesis of **AIPA-Gallic acid**

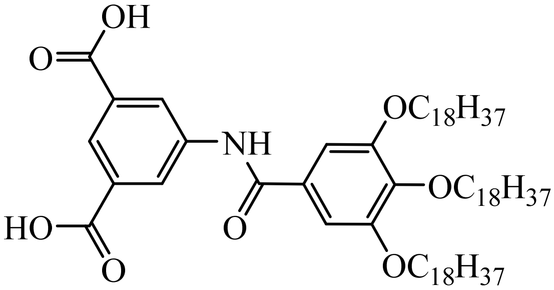

**AIPA-Gallic acid**

Potassium hydroxide (1.98 g, 354 mmol) was dissolved in 250 mL of 2-ethoxyethanol. To this solution was added AIPA-Gallic acid (3.96 g, 354 mmol). The resulting mixture was refluxed for 4 hours. After cooling down to room temperature, and concentration under reduced pressure, the resulting dense solution was acidified by hydrochloric acid (1M) until pH= 5. The white formed precipitate was filtered and washed with distilled water (2x300 mL). The resulting powder was dissolved in hot methanol and let to cool down in the fridge for 24 hours upon which period a white precipitate was formed. The resulting powder was filtered and washed with cold ethanol.

White powder, 2.80 g, Yield 83%, m.p.: 78° C, FT-IR (KBr) ν ($cm^{-1}$): 3600-3200, 2918, 2850 ($CH_x$), 1716 (C=O), 1649 (C=O), 1585, 1535, 1501 (C=C), 1280, 1233, 1122, 756, 720; $^1$H-NMR (500 Mhz, $CDCl_3$) δ (ppm): 7.27 (s, 2H), 6.92 (s, 1H), 6.54 (s, 2H), 4.1-3.8 (m, 6H), 3.47 (br s, 1H), 1.9-1.7 (m, 6H), 1.5-1.4 (m, 6H), 1.3-1.1 (m, 84H), 0.9-0.8 (m, 9H); anal. ($C_{69}H_{119}NO_8$): calcd C: 75.98, H: 11.00, N: 1.28%; found C: 75.79, H: 10.81, N: 1.46%.

## Steady Rheology experiments:

In Figure 1, the typical variation of viscosity, η, versus shear rates, for samples at different temperatures is given for the systems oil with and without additive It is immediately evident that different systems have different rheological behaviours. A Newtonian behaviour is apparently observed for oils without additive, where the viscosity is low and independent of shear rates. For the oils with the additive, the viscosity decreases at higher shear rates such as in a shear-thinning fluid, also known as a pseudoplastic system. This behaviour is similar to that found for gels Malkin, A.Y.; Derkach, S.R.; Kulichikhin, V.G. Rheology of Gels and Yielding Liquids. *Gels* **2023**, *9*, 715. https://doi.org/10.3390/gels9090715. The shear thinning behaviour appears increasingly pronounced as the surfactant concentration increases.

**a)**

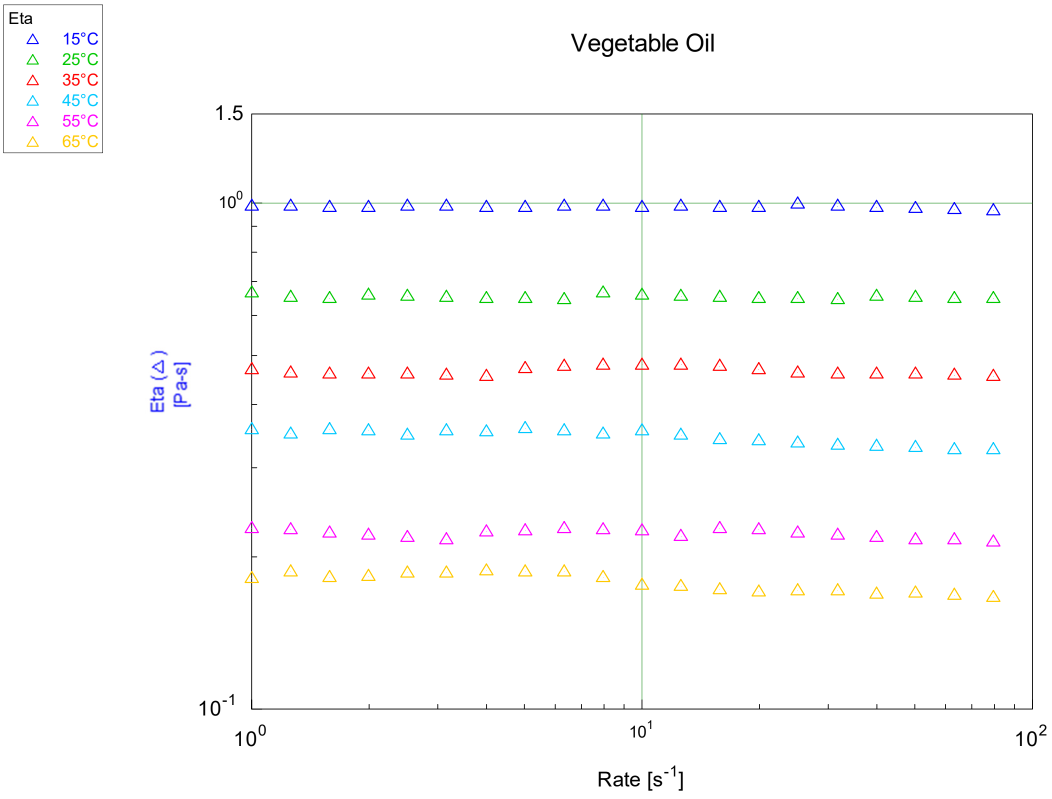

**b)**

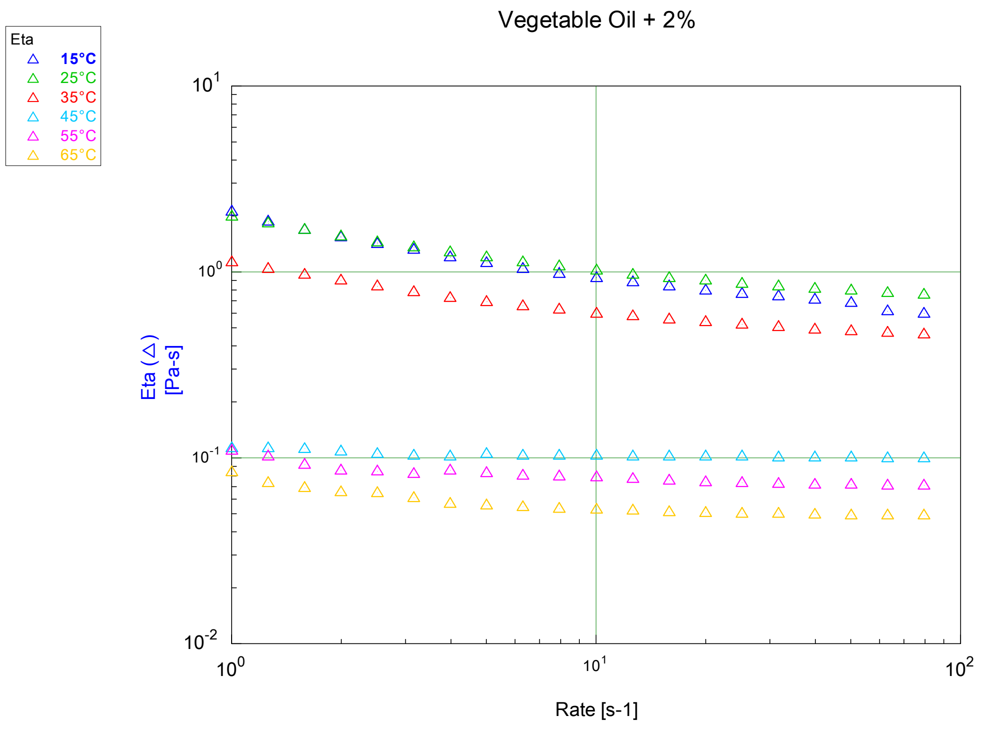


**c)**

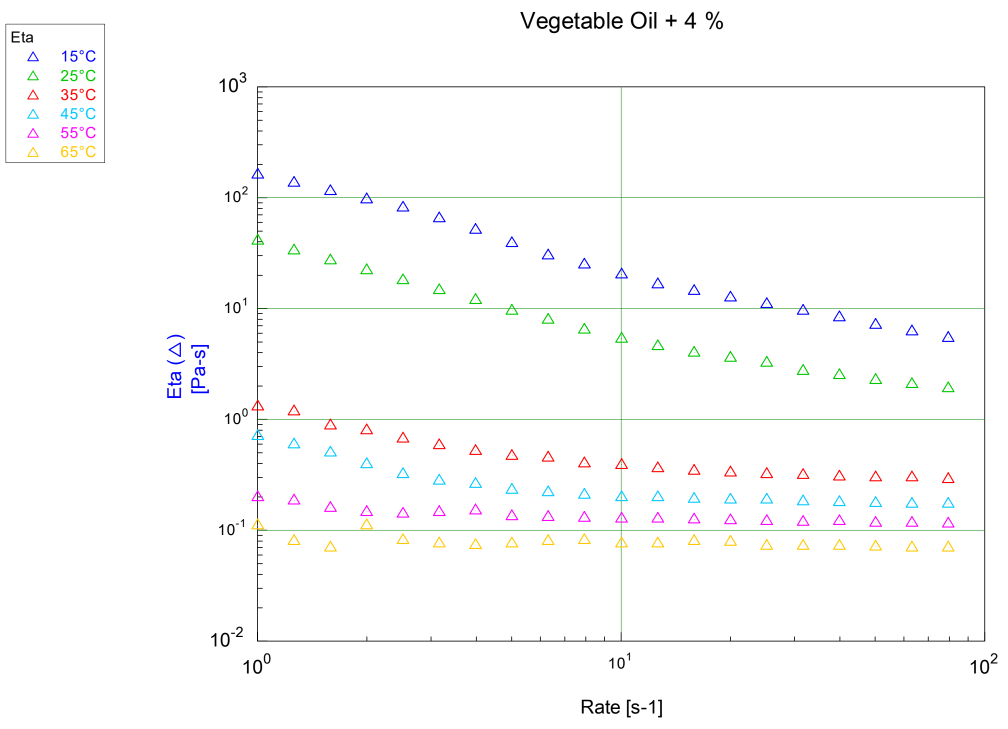


Figure S1. Flow curves of the Vegetable oil at various temperatures: without additive (a), with 2% w/w AIPA–gallic acid (b), and with 4% w/w AIPA–gallic acid (c).

a)

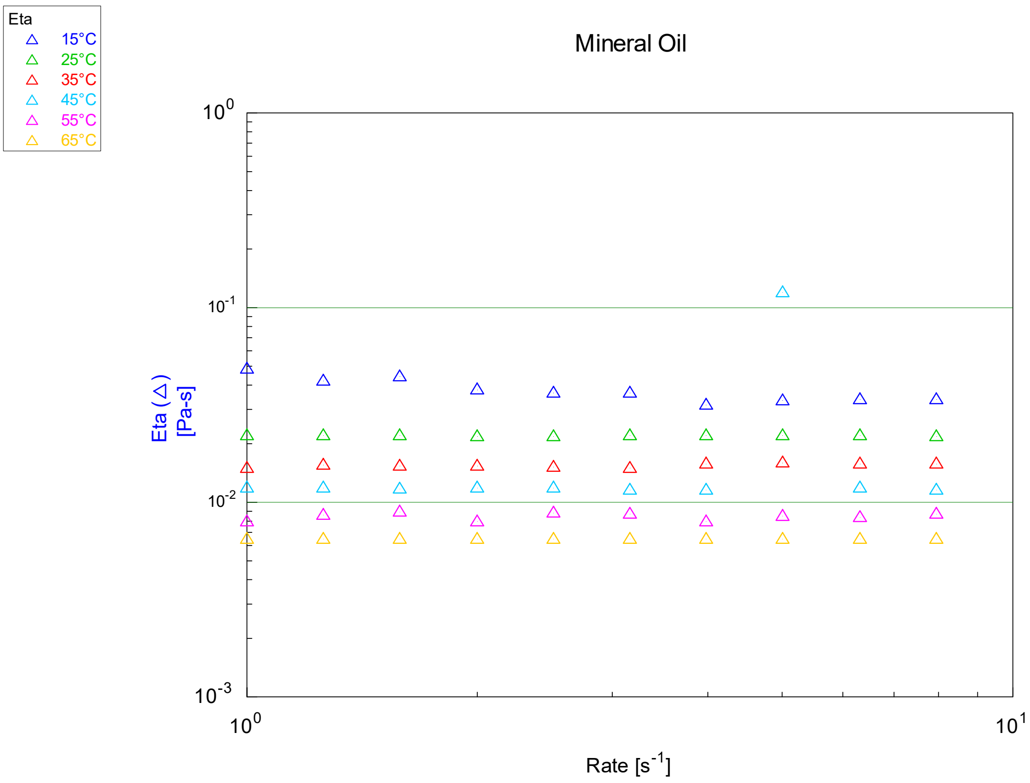
Eta
15°C
25°C
35°C
45°C
55°C
65°C
Mineral Oil
10^0
10^-1
10^-2
10^-3
Eta (Δ)
[Pa-s]
10^0
10^1
Rate [s^-1]

b)

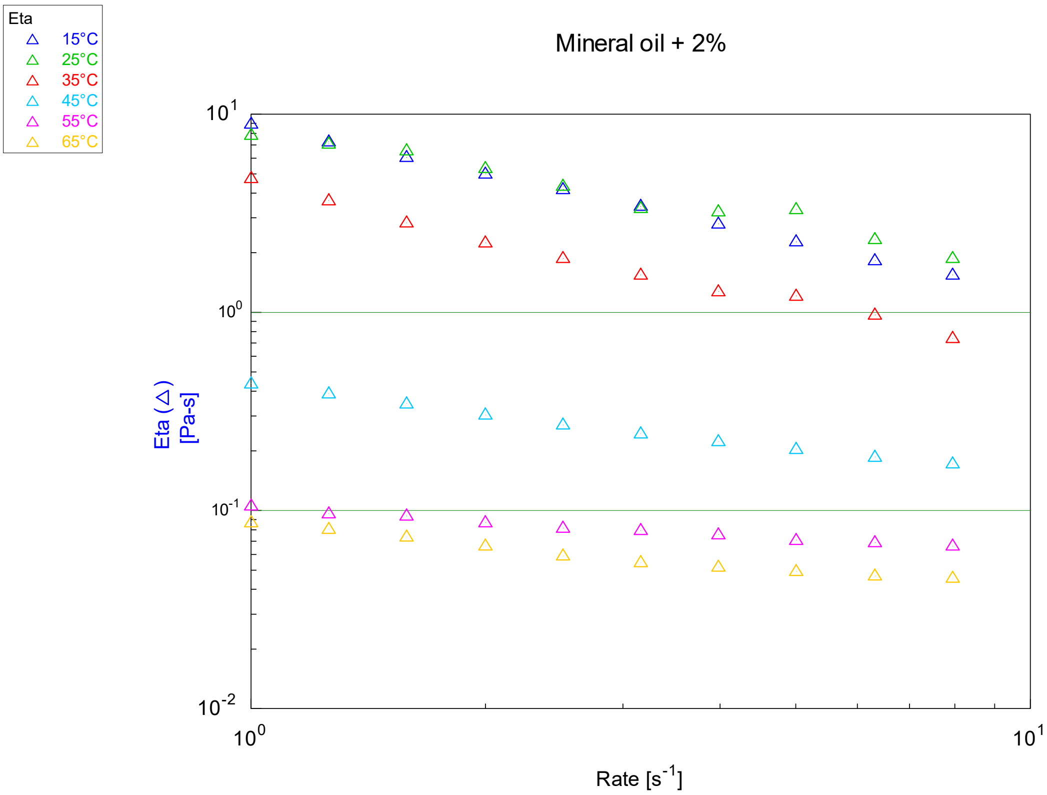
Eta
15°C
25°C
35°C
45°C
55°C
65°C
Mineral oil + 2%
10^1
10^0
10^-1
10^-2
Eta (Δ)
[Pa-s]
10^0
10^1
Rate [s^-1]

**c)**

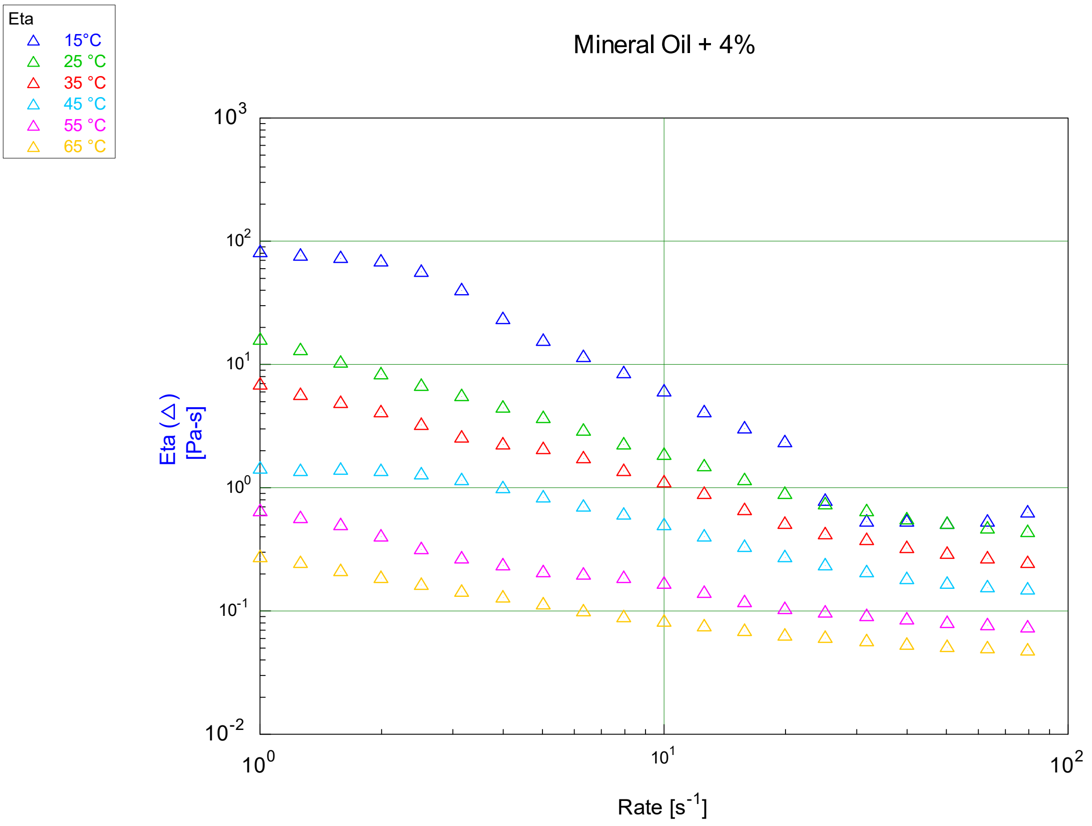


Figure S2. Flow curves of the Mineral oil at various temperatures: without additive (a), with 2% w/w AIPA–gallic acid (b), and with 4% w/w AIPA–gallic acid (c).
